# Supplementary material for: Comprehensive Annotation and Functional Exploration of MicroRNAs in Lettuce
Source: Front Plant Sci. 2021 Dec 24;12:781836. doi: 10.3389/fpls.2021.781836 (PMC8739914; doi:10.3389/fpls.2021.781836)
Supplement: Supplementary file 3 [file Data_Sheet_9.ZIP › GSM2230751.plot/Lsa-miR6114_Lsat_1_v5_gn_5_158001.1_4479_TPlot.pdf]

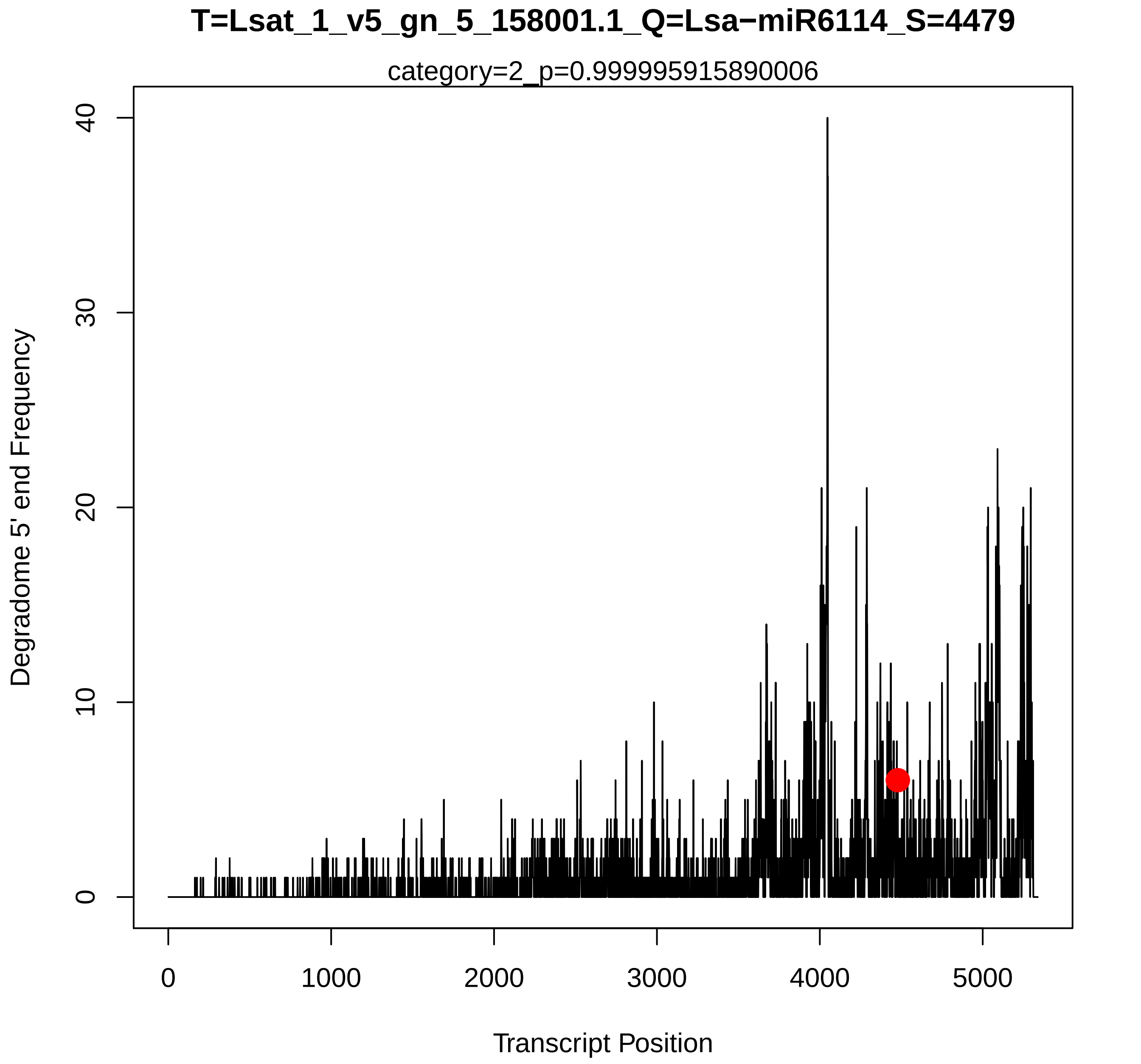
T=Lsat_1_v5_gn_5_158001.1_Q=Lsa−miR6114_S=4479
category=2_p=0.999995915890006
Degradome 5' end Frequency
Transcript Position
0
10
20
30
40
0
1000
2000
3000
4000
5000
